# Bedside conventional tracheostomy^☆^^☆☆^

**DOI:** 10.1016/j.bjorl.2025.101602

**Published:** 2025-05-29

**Authors:** Giancarlo Artese Araujo, Antonio Augusto Tupinambá Bertelli, Helvécio de Resende Urbano Neto, Marianne Yumi Nakai, Caroline Schmiele Namur, Marcelo Benedito Menezes, Antonio José Gonçalves

**Affiliations:** Santa Casa de São Paulo, Faculdade de Ciências Médicas, São Paulo, SP, Brazil

**Keywords:** Tracheostomy, Intensive care, Intraoperative complications, Surgical procedures, Elective, Surgicenters

## Abstract

**Objective:**

To describe the open experience of Head and Neck Surgery division of Santa Casa in São Paulo with bedside tracheostomy.

**Methods:**

A retrospective study of database tracheostomies was performed between 2013 and 2016. Then, age, gender, and main complications were analyzed.

**Results:**

Analysis of 598 patients shows 9 cases (1.50%) of complications, bleeding was the cause in 6 cases (1.0%), 1 postoperative death (0.16%), 1 accidental decannulation case (0.16%) and 1 lesion in the posterior wall of the trachea (0.16%). It is possible to accomplish bedside open tracheostomy with safety.

**Level of evidence:**

Level IV.

## Highlights

• Tracheostomy is a frequent procedure in Intensive Care Units.
• The ideal time to perform a tracheostomy is controversial but some laryngologists recommend it after 3 days of intubation, although most consider a limit of 2–3 weeks.
• Tracheostomy at the bedside, with local anesthesia and sedation, is a safe procedure with results similar to those performed in an operating room, with lower costs, avoiding transporting critical patients to the operating room.
• In this study with 598 patients, only 1.50% of the patients had complications and the most part was bleeding (six patients).

## Introduction

Tracheostomy is a very frequent procedure in Intensive Care Units (ICU), as it is used for patients who require prolonged mechanical ventilation. Its benefits include reducing the risk of laryngeal damage, aiding ventilation and pulmonary hygiene, improving patient comfort and oral care, facilitating nursing care, weaning from mechanical ventilation and oral feeding.^1^, ^2^ There is no fixed rule regarding the time an endotracheal cannula can be left. Some laryngologists recommend conversion to tracheostomy after 3 days of intubation, although most consider a limit of 2–3 weeks.^1^ Although it is a controversial issue, data in the literature have already indicated that early tracheostomy led to a significant reduction in the length of ICU stay and hospital stay, mechanical ventilation time and the incidence of ventilator-associated pneumonia.^2^, ^3^ Other indications of tracheostomy include chronic aspiration, acute airway obstruction secondary to facial and laryngeal trauma, deep neck space infections or perioperative tracheostomy during radical cancer ablation.^1^

The main complications related to tracheostomy are bleeding, wound infection, tracheoesophageal fistula and tracheo-innominate artery fistula.^4^ Tracheostomy at the bedside, with local anesthesia and sedation, is a safe procedure with results similar to those performed in a operating room, avoiding the transport of critical patients to the operating theater, with lower costs.^5^

Both open and percutaneous tracheostomy techniques have advantages and disadvantages. Complications such as paratracheal insertion, tracheal laceration, technical difficulties, and pneumothorax tend to be more frequent in the percutaneous procedure, while inflammation and wound infection are more commonly reported in the open approach.^6^

The objective of this study is to describe the bedside experience of the Head and Neck Surgery Division of Santa Casa de São Paulo with open tracheostomies performed outside the operating room environment from 2013 to 2016.

## Methods

This is a retrospective study that analyzed the database of 598 patients from the Division of Head and Neck Surgery of Irmandade da Santa Casa de São Paulo, from August 2013 to October 2016, submitted to open tracheostomy, at the bedside in the Intensive Care Unit or emergency unit beds.

Surgeries were performed by residents of general surgery and head and neck surgery, under the guidance of a head and neck surgeon trained and experienced in the procedure. The technique performed was open sub-thyroid-tracheostomy performed at the bedside with transverse or longitudinal incision in the skin and transverse tracheotomy on the second or third tracheal membrane under local anesthesia and sedation.

## Results

A sample of 598 patients was obtained. The mean age of the study population was 58-years-old, with a predominance of males (62%) (Table 1). The main indication for tracheostomy was prolonged orotracheal intubation (99.6%). Only nine of the patients submitted to tracheostomy suffered complications (1.50%). Six patients were described with bleeding, one with accidental decannulation, one with tracheal injury and one died (due to hemodynamic instability during the procedure) (Table 2).

**Table 1:** Epidemiological characteristics of patients submitted to tracheostomy.

Variables	n (%) or Mean
Age	58
Gender	
Male	373 (62)
Female	225 (38)

**Table 2:** Complications related to bedside tracheostomy.

Complications	n (%)
Total	9 (1.50)
Bleeding	6 (1.00)
Death	1 (0.16)
Accidental decannulation	1 (0.16)
Tracheal posterior wall injury	1 (0.16)

## Discussion

Although tracheostomy was performed in intensive care critical patients, there were few complications related to the procedure, even performed by surgeons in training, under the supervision of a specialist, demonstrating that the difficulties related to the site were not significant. The complication rate observed in the study was lower than other teaching hospitals, where tracheostomies were also performed by resident physicians under the supervision of specialists. In the Perfeito et al. study, in which the open tracheostomies were performed at the bedside in an Intensive Care Unit at Hospital São Paulo, there was a complication rate of 5.4%, 2.7% due to bleeding and 2.7% due to wound infections.^7^ Terra et al. reported a similar complication rate of 4.3% at Hospital das Clínicas of the University of São Paulo.^8^

The greatest benefits of performing bedside tracheostomy are lower cost and lower rates of complications related to the transport of the critical patient to the operating center. Many of these patients have injuries that make their mobilization very dangerous, or they have venous accesses (sometimes vasoactive drugs), drainages or more invasive monitoring that can be displaced during transportation, complicating or threatening their lives. Tracheostomy at the bedside can be performed sometimes earlier since it does not depend on the availability of a room in the operating theater, often overloaded.^7^ Available evidence from randomized controlled trials including critical patients tends to show that percutaneous techniques are faster and associated with lower infection rate and wound inflammation, but related to greater technical difficulty.^9^, ^10^, ^11^ Other complications, such as intra and postoperative bleeding, accidental decannulation, tracheal tube obstruction and death were not statistically significant in some studies.^10^, ^11^ When percutaneous technique is associated with dilatation procedures such as MDT (Multiple Dilatation Tracheostomy) and SSDT (Single-Step Dilatation Tracheostomy), there is a lower risk of bleeding and technical difficulties, and its use in critical patients is therefore preferable.^9^ In a meta-analysis of Putensen et al., the major bleeding rate was 1.34% for the percutaneous procedure and 4% for the open procedure, with a statistically significant difference. However, in this study, the risk of bleeding was 1.0%.

Terra et al. also analyzed the cost of procedures performed in the different hospital settings: US$ 253 for the open technique and US$ 494 for the percutaneous technique in the ICU; and US$ 496 for the open technique at the operating room.^8^ This analysis is important when considering the difficulty, in terms of costs, of performing the tracheostomy in the public health system. Besides the cost, percutaneous tracheostomy usually requires the availability of a professional to perform bronchoscopy or ultrasonography.

Also, it is worth noting the importance of performing the open procedure when training residents, which is the main objective of teaching hospitals.

In conclusion, this study corroborates the concept that open bedside tracheostomy is a viable, and safe, even in the Intensive Care environment, since the minimum requirements for materials, safety, and standardization of the surgical technique are respected.

## Funding

None.

## Declaration of competing interest

The authors declare no conflicts of interest.
